# Factors associated with the effectiveness of oral health promotion in the Family Health Strategy

**DOI:** 10.1590/1807-3107bor-2025.vol39.096

**Published:** 2025-09-15

**Authors:** Suyene de Oliveira PAREDES, Franklin Delano Soares FORTE, Edson Hilan Gomes de LUCENA, Andreza Cristina de Lima Targino MASSONI, Maria Helena Rodrigues GALVÃO

**Affiliations:** (a) Universidade Federal da Paraíba – UFPB, Health Sciences Center, Post-graduation Program in Dentistry, João Pessoa, PB, Brazil.; (b) Universidade Federal de Pernambuco – UFPE, Vitoria Center Academic, Vitória de Santo Antão, PE, Brasil.

**Keywords:** Health Promotion, Oral Health, Primary Health Care, Health Evaluation

## Abstract

This study investigated associations between sociodemographic and professional profiles, work-related factors, and the effectiveness of oral health promotion strategies implemented by dentists in the Family Health Strategy. A cross-sectional study was conducted with 211 dentists working in Oral Health Teams within the Family Health Strategy in the state of Paraíba, Brazil. Data were collected online using a validated instrument. A matrix encompassing the core values and pillars of health promotion was employed to evaluate the effectiveness of oral health promotion strategies. Data were analyzed using Poisson regression (p < 0.05), and all analyses were performed in Stata, version 14. Greater effectiveness of oral health promotion strategies was associated with mixed Oral Health Teams (urban and rural coverage) (PR = 1.54; 95%CI: 1.154–2.076; p = 0.003) and with dentists under temporary contracts (PR = 1.67; 95%CI: 1.240–2.250; p = 0.001). The effectiveness of oral health promotion strategies was associated with work-related factors. Evaluations of oral health promotion practices are essential to support improvements in oral health management. The findings highlight the need to strengthen these practices through professionals who, in addition to having a defined employment relationship and a structured work process, value oral health promotion as a key component of care.

## Introduction

The concept and implementation of health promotion have evolved through successive international conferences on health promotion, likely influenced by the sociopolitical and health contexts of each period.^1^ There is a global consensus that the health and social well-being of individuals and communities are shaped by a wide range of factors beyond the health system. Consequently, health promotion must be integrated into all public policies to achieve meaningful improvements in health outcomes.^1,2^

In Brazil, health promotion was established to enhance quality of life and reduce health risks and vulnerabilities. More recently, it has gained prominence in public health policy.^3^ National guidelines encourage research to assess the efficiency, efficacy, effectiveness, and safety of health promotion actions, a goal further supported by the revised and updated National Health Promotion Policy, which aims to inform evidence-based decision-making.^3,4^ Effectiveness refers to whether an intervention achieves its intended purpose under real-world, uncontrolled conditions.^5,6^ This assessment depends on the perceptions of those involved and the timing of the evaluation^6^. Thus, its results should be interpreted as indicative rather than conclusive evidence of effectiveness.^6^

The National Oral Health Policy (PNSB), legally established within the Unified Health System (SUS), guarantees oral health as a right for all Brazilians.^7^ Furthermore, PNSB emphasizes the role of evaluation as part of the planning and programming process, with a focus on health surveillance and the monitoring of actions carried out within local territories.^7^

Since its implementation in 2004, the PNSB has expanded access to public oral health services across Brazil.^8,9^ Nevertheless, the model continues to exhibit inequalities,^8^ and achieving integrated care remains a challenge for health system management.^9^ In addition, individual and territorial factors significantly influence oral health outcomes.^10-12^ Individuals who have never visited a dentist are more likely to come from lower socioeconomic backgrounds, be illiterate, and self-identify as Black.^10,11^ Greater reliance on surgical or emergency procedures—often resulting in tooth extractions—has been associated with advanced age, presence of toothache, self-reported Black race or color, and residence in the Northeast region.^11,12^

The strengthening of health promotion practices helps to reduce inequalities and enhance well-being and quality of life.^3,4,6^ Evaluating Oral Health Promotion (OHP) initiatives is therefore crucial, particularly considering the growing body of evidence supporting the positive impact of these practices on oral health outcomes.^13^ Studies that monitor OHP actions,^13,14^ especially those grounded in theoretical models encompassing the core pillars and values of health promotion,^15,16^ have shown that the South and Southeast regions of Brazil achieve the best performance scores.^14^ Brazilian capitals with the most favorable health ratings also demonstrate the greatest potential for promoting health.^13^ In contrast, the lowest performance was observed in capitals that showed limited improvement in decayed, missing, filled teeth indices among adolescents.^13^

Evaluating the effectiveness of OHP initiatives implemented by Oral Health Teams (OHTeams) in the Family Health Strategy (FHS) may generate much-needed knowledge to support oral health management. The FHS is a health care model designed to reorganize primary care in Brazil, with a focus on prevention and health promotion.

This study was guided by the hypothesis that sociodemographic characteristics, professional profiles, and work-related factors of OHTeam dentists may influence the effectiveness of OHP strategies, considering that team structure,^17^ regional conditions,^14,17,18^ and training or continuing education^19^ have all been shown to affect team performance. Accordingly, this study aimed to investigate the associations between sociodemographic and professional profiles, work-related factors, and the effectiveness of oral health promotion strategies implemented by FHS dentists.

## Methods

### Study design and location

This cross-sectional epidemiological study was designed and conducted in accordance with the Strengthening the Reporting of Observational Studies in Epidemiology (STROBE) guidelines. The state of Paraíba comprises 223 municipalities, has a land area of 56,467.24 km^2^, and had an estimated population of 3,974,495 inhabitants in 2022.^20^ The Human Development Index (HDI) for 2021 was 0.698^20^. In August 2021,^21^ the population coverage of the OHTeam within the FHS was 89.32%. In terms of health regionalization, the state is divided into three health macro-regions and subdivided into 16 health regions.^22^

Socioeconomic indicators such as life expectancy, illiteracy rate, the percentage of the population in extreme poverty, vulnerability to poverty, and per capita income are lower in Paraíba compared to the national averages.^23^ Additionally, according to the federal government’s Single Registry for Social Programs (CadÚnico), 36.48% of the population lives without an adequate water supply, 47.39% without sanitation, and 27.72% without waste collection.^23^

Given these unfavorable sociodemographic conditions^23^ and the high prevalence of oral health problems in the state,^24-26^ the evaluation of OHP practices in Paraíba is critical. Epidemiological data show a high prevalence of dental caries, toothache, and tooth loss across all age groups,^24-26^ negatively impacting quality of life.^26^ Furthermore, the performance of the OHTeam in primary care has been considered insufficient in terms of treatment resolution, suggesting that primary oral health care services face challenges in completing the treatment plans.^27^

### Participants and eligibility criteria

The target population consisted of 1,400 dentists working in OHTeams in the state of Paraíba as of August 2021.^21^ Participants were selected using non-probabilistic snowball sampling, in which eligible individuals referred others.^28^ In cases where no referral was provided, an active search was conducted.

Dentists were eligible if they had been working in the same OHTeam for at least one year prior to the onset of the COVID-19 pandemic. This inclusion criterion was established considering the restrictive measures imposed during the pandemic, which led to reduced individual dental care and the suspension of collective procedures.^29^ Dentists who had been away from work for six months or more for any reason were excluded.

A total of 781 dentists were invited to participate. Of these, 12 were excluded because they worked exclusively in private or secondary care settings, and 6 were excluded based on insufficient work experience. Among the 763 eligible individuals, 522 did not respond or declined to participate. The final sample comprised 211 dentists, resulting in a response rate of 28%.

### Outcome and covariates

The outcome variable was the effectiveness of OHP strategies. Covariates included sociodemographic and professional characteristics of the dentists, as well as work-related factors within the OHTeams. Sociodemographic covariates comprised sex (male or female), marital status (single, married, or divorced), parenthood (yes or no), and age (categorized into terciles: ≤ 28, 29–36, or ≥ 37 years). Professional profile variables included residence and employment in the same municipality (yes or no), time since graduation (≤ 4, 5–10, or ≥ 11 years), and concurrent employment in another public or private setting (yes or no). Family income (≤ 4, 5–6, or > 6 minimum wages) and overall work experience (≤ 3, 4–9, or ≥ 10 years) were also categorized into tertiles.

Work-related covariates within the OHTeam included time working in the OHTeam (≤ 2, 3–5, or ≥ 6 years), remuneration (≤ 2, 3–4, or ≥ 5 minimum wages), team type based on coverage area (urban, rural, or both), and employment relationship according to the National Register of Health Establishments—categorized as a statutory system, CLT system (Consolidation of Labor Laws), or temporary contract)^30^. Additional covariates were weekly workload (total hours worked in the OHTeam) and health macro-region (i.e., the municipality where the dentist worked), classified as first (João Pessoa), second (Campina Grande), or third (Sertão and Alto Sertão) macro-region.^22^

The effectiveness of the OHP strategies was assessed using a validated instrument developed by Kusma, Moysés, and Moysés.^15,16^ This tool comprises a matrix of descriptors divided into three dimensions: oral health, health-related public policies, and human and social development. A total of 23 indicators were evaluated. Each indicator was rated on a five-point Likert scale, ranging from 1 (does not address OHP issues) to 5 (fully addresses OHP issues). Total scores could range from 23 to 115, with higher scores indicating greater potential to promote oral health. The effectiveness of OHP strategies was then categorized as either lower potential (score: 23–74), when the actions did not incorporate the core pillars and values of OHP in primary health care, or higher potential (score: 75–155) when they did^16^.

### Data collection

Data were collected between August 2021 and May 2022 by a single researcher. However, participants were asked to report on their professional experience prior to the Covid-19 pandemic. Initial invitations were sent to eligible dentists via WhatsApp®. This platform was also used to send a link granting access to an interactive PDF, which provided information on the study’s eligibility criteria and directed participants to the informed consent form. Upon consent, participants gained access to a virtual questionnaire developed using Google Forms®^31^. The questionnaire consisted of two sections: the first addressed sociodemographic and professional characteristics of the dentists and was reviewed by three experts; the second contained the matrix of descriptors assessing OHP in primary health care.^15,16^ Based on a pilot study, the average completion time for the questionnaire was estimated at 18 minutes. Additionally, invitation and participation links were sent via email, when available. If no response was received to the initial invitation, a single follow-up was made via WhatsApp or email.

Sampling was conducted using the snowball technique.^28^ Initially, participants meeting the inclusion criteria were directly invited. Those who agreed were then asked to refer other professionals who also met the research criteria. In the absence of referrals, an active search was conducted using contact lists provided by municipal health departments.

### Statistical analysis

Descriptive statistics were used to characterize the sample. Simple and multiple Poisson regression models with robust variance estimation were employed to examine associations between the outcome (effectiveness of oral health promotion strategies) and covariates. These models estimated prevalence ratios (PR), 95% confidence intervals (95%CI), and p-values. Unadjusted bivariate analyses were conducted first; covariates with p ≤ 0.20 were included in the adjusted multivariate model, taking into account their hierarchical position. In the final model, associations were considered statistically significant at p < 0.05. Model fit was assessed using deviance values (−2 log-likelihood). Analyses were performed using STATA version 14.

### Ethical aspects

This study was conducted in accordance with the Declaration of Helsinki and approved by the relevant research ethics committee (approval numbers: 4.724.462 and 5.025.305). Written informed consent was obtained from all participants.

## Results

A total of 211 dentists working in OHTeams in the state of Paraíba were included. Most participants were women (n = 141, 66.8%), single (n = 100, 48.5%), did not have children (n = 122, 57.8%), lived in the same municipality where they worked (n = 120, 56.9%), and held additional dental jobs in other settings (n = 147, 69.7%) (Table 1).

**Table 1:** Sociodemographic and professional characteristics of the sample. Paraíba, Brazil, 2022.

Variable	n	%
Sex (n = 211)		
Female	141	66.8
Male	70	33.2
Age (years) (n = 211)		
< 28	72	34.1
29–36	70	33.2
≥ 37	69	32.7
Marital status (n = 206)		
Single	100	48.5
Marriage	92	44.7
Divorced/separated	14	6.8
Children (n = 211)		
Yes	88	41.7
No	122	57.8
Family income (MW) (n = 211)		
< 4	75	35.5
5–6	66	31.3
≥ 6	70	33.2
Resides in municipality of work (n = 211)		
Yes	120	56.9
No	91	43.1
Time since graduation (years) (n = 211)		
< 4	83	39.3
5–10	61	28.9
≥ 11	67	31.8
Time in professional practice (years) (n = 211)		
< 3	76	36.0
4–9	65	30.8
≥ 10	70	33.2
Other jobs (n = 211)		
Yes	147	69.7
No	64	30.3

MW: Minimum wage for 2021 (R$ 1,100.00) to 2022 (R$ 1,212.00).

Regarding work-related factors in the current OHTeam, 40.8% (n = 86) of participants reported a work experience of ≤ 2 years. Most earned between 3 and 4 minimum wages (n = 137, 64.9%), while 32.2% earned ≤ 2 minimum wages. Slightly more than half were employed under statutory or CLT systems (n = 109, 52.4%). Most dentists were in urban teams (n = 106, 50.2%), had a 40-hour weekly workload (n = 191, 90.5%), and were based in the third health macro-region of Paraíba (n = 102, 48.3%) (Table 2). Ninety-eight dentists (46.4%) demonstrated a higher potential to promote oral health through OHP, while 113 (53.6%) exhibited lower potential (Table 2). The mean total score was 73.05 (± 21.49). The final model revealed that greater potential for promoting oral health was associated with working in both urban and rural areas (PR = 1.54, 95%CI: 1.15–2.08) and holding a temporary contract (PR = 1.67, 95%CI: 1.24–2.25) (Table 3).

**Table 2:** Work-related characteristics of the sample within the eFHS and OHTeams. Paraíba, Brazil, 2022.

Variable	n	%
Time working in the eOH (years) (n = 211)		
< 2	86	40.8
3–5	58	27.5
≥ 6	67	31.8
OHTeam remuneration (MW) (n = 211)		
< 2	68	32.2
3–4	137	64.9
≥ 5	6	2.8
Type of OHTeam based on catchment area (n = 211)		
Urban	106	50.2
Rural	37	17.5
Mixed	68	32.2
Employment relationship (n = 208)		
Statutory/CLT	109	52.4
Temporary employee	99	47.6
Weekly working hours (n = 211)		
20	6	2.8
32	14	6.6
40	191	90.5
Health macro-region (n = 211)		
1^ST^ João Pessoa	59	28.0
2^ND^ Campina Grande	50	23.7
3^rd^ Sertão and Alto Sertão	102	48.3
Effectiveness of OHP practices		
Greater potential	98	46.4
Lower potential	113	53.6

MW: Minimum wage.

**Table 3:** Sociodemographic, professional, and work-related factors associated with the effectiveness of oral health promotion strategies. Paraíba, Brazil, 2022.

Variable	Unadjusted PR		Adjusted PR	
	n (95% IC)	p-value	n (95% IC)	p-value
Sex				
Female	0.93 (0.616-1.416)	0.750		
Male	1			
Age (years)				
< 28	1			
29–36	1.09 (0.670-1.787)	0.717		
≥ 37	1.14 (0.703-1.862)	0.587		
Marital status				
Single	1			
Marriage	1.27 (0.838-1.930)	0.257		
Divorced/separated	1.21 (0.547-2.718)	0.627		
Children (n = 211)				
Yes	1	0.313		
No	0.81 (0.548-1.212)			
Family income (MW)				
< 4	1.10 (0.692-1.773)	0.668		
5–6	0.92 (0.558-1.541)	0.773		
> 6	1			
Resides in municipality of work				
Yes	1.09 (0.734-1.645)	0.644		
No	1			
Time since graduation (years)				
< 4	1			
5–10	0.77 (0.473-1.263)	0.304		
≥ 11	0.81 (0.510-1.304)	0.397		
Time in professional practice (years)				
< 3	1			
4–9	0.72 (0.443-1.180)	0.195		
≥ 10	0.77 (0.485-1.238)	0.288		
Other jobs				
Yes	0.98 (0.721-1.350)	0.934		
No	1			
Time working in the eOH (years)				
< 2	1			
3–5	0.78 (0.485-1.281)	0.338		
≥ 6	0.71 (0.439-1.146)	0.161		
OHTeam remuneration (MW)				
< 2	1.00 (0.307-3.255)	1.000		
3–4	0.89 (0.279-2.838)	0.845		
≥ 5	1			
Type of OHTeam (catchment area)				
Urban	1		1	
Rural	0.95 (0.521-1.748)	0.881	1.00 (0.627-1.613)	0.980
Mixed	1.55 (1.016-2.390)	0.042	1.54 (1.154-2.076)	0.003
Employment relationship				
Statutory/CLT	1		1	
Temporary employee	1.70 (1.137-2.569)	0.010	1.67 (1.240-2.250)	0.001
Weekly working hours				
20	1			
32	0.71 (1.707-2.988)	0.645		
40	0.94 (2.983-2.977)	0.919		
Health macro-region				
1^ST^ João Pessoa	1			
2^ND^ Campina Grande	1.05 (0.614-1.806)	0.850		
3^rd^ Sertão and Alto Sertão	0.92 (0.579-1.490)	0.762		

CI: Confidence interval; pr: Prevalence ratio; PR: Poisson regression.

## Discussion

Evaluating the effectiveness of health promotion programs can be complex.^14,32^ However, when appropriate tools and methodologies are applied to different contexts and territories, they can help identify both health-related challenges and weaknesses in work processes.^32^ From this perspective, the use of an evaluation framework grounded in the pillars (participation, equity, and sustainability) and values (autonomy, empowerment, comprehensiveness, intersectionality, and governance) of health promotion^15,16^ proved to be a valuable tool for assessing OHP strategies implemented by dentists working in the FHS in Paraíba.

A higher potential for promoting oral health was observed in 46.4% of the teams, while 53.6% achieved less satisfactory results. The overall mean score was 73.05. In comparison, another study reported national and Northeast regional averages of 71.30 and 69.25, respectively. João Pessoa stood out as the Brazilian capital with the highest level of effectiveness, with a mean score of 81.87.^14^

In this study, two factors were significantly associated with higher oral health promotion potential. First, dentists working in both urban FHS units (located in municipal centers) and rural support units were more likely to perform better. This mixed coverage model likely supports broader and more diverse practices.^33,34^ As a result, mixed teams may be better positioned to develop care processes that account for the specificities of rural contexts—fostering dialogue and encouraging practices that respect cultural differences, traditional knowledge, and local lifestyles.^35^

Regarding the OHTeam modalities, one study reported that Modality II—composed of a dentist, an oral health technician, and an additional oral health technician or oral health assistant—along with location in Brazil’s South and Southeast regions, was associated with better work process performance, particularly in action planning, health promotion, and comprehensive care.^17^ In contrast, the present study found that the municipality (i.e., health macro-region) where the dentist worked did not significantly influence the outcome.

The employment relationship, however, was associated with the effectiveness of the oral health promotion strategies. In this study, the statutory system and the CLT system were grouped due to their greater job stability and legal protections when compared with temporary contracts. Interestingly, it was the temporary contracts that were associated with greater potential for implementing oral health promotion strategies. This finding contrasts with prior research indicating that the statutory system contributed to better performance among physicians working in eFHS teams.^36^

The precarious nature of dental work must be acknowledged, considering that it is shaped by unstable contracts, low pay, high productivity demands, pressure to perform unnecessary procedures, and restrictions on professional autonomy due to the commercial interests of dental plans and popular private clinics. These conditions may lead to insecurity in private practice settings,^37^ thereby improving performance among dentists working in the public sector.

It is also worth noting that this study did not observe any collinearity between the employment relationship and either the dentist’s age or time since graduation. In the current context, dentistry education programs in Brazil have been progressively aligning with updated curricular guidelines aimed at developing health promotion skills and capabilities.^38^

Although this study focused exclusively on dentists, the average weekly workload was reported to be less than 40 hours. According to Technical Note 19/2022 issued by the Ministry of Health, OHTeams linked to FHS Teams (eFHS) are expected to work a minimum of 40 hours per week.^39^ However, the requirement allows more flexibility for newly formed teams, where two OHTeams—each with a 20-hour weekly workload—can be assigned to a single eFHS. Nevertheless, it is important to emphasize that this flexibility was formalized in 2022, while the present study is based on pre-pandemic data. Moreover, dentists who reported working 32 hours per week may have factored in a weekly day off occasionally granted by the municipal administrators.^40^

This study used a validated and applicable instrument, taking into account the restrictive measures imposed during the Covid-19 pandemic. However, two main factors limited the sample size. First, some dentists were not formally recruited. Second—and possibly as a consequence of the first—there was high professional turnover. Many professionals had not been working for at least a year before the onset of the pandemic, likely due to mayoral elections held in January 2021. As a result, sample loss in this study prevented the formation of a representative sample, thus limiting the generalizability of the findings, given the size and diversity of the state of Paraíba.

Additionally, the snowball sampling technique introduced a potential participation bias, attributable to limited control over how individuals selected members of their personal networks.^31^ To mitigate this, the initial contacts were carefully chosen. It is important to note that snowball sampling proved to be a viable and scientifically rigorous strategy during the COVID-19 pandemic^31^—a time when social distancing restricted access to professionals such as dentists.

Services provided to communities, especially those within primary health care, must be continuously monitored and evaluated to improve interventions and inform the development of public health policies that promote universality, comprehensiveness, and a community-centered approach.^6,15^ Although this study offers a snapshot of OHP effectiveness, the results indicate that the success of oral health promotion strategies may be influenced by factors within the OHTeams.

## Conclusions

Since dentists with temporary contracts demonstrated greater effectiveness in promoting oral health, efforts should be directed toward improving the performance of permanent professionals within OHTeams. The findings of this study can support oral health management strategies based on the matrix of indicators applied. Future evaluation studies with alternative methodological designs are needed to explore aspects that cross-sectional studies cannot fully explain. These results offer a critical reflection on the sustainability of current practices, an outcome that depends on professionals who not only have stable employment and structured work processes but also value oral health promotion as a central care strategy.

This study offers a novel perspective on labor relations in the context of health promotion. It suggests that future research should incorporate variables such as job insecurity, employment arrangements, professional turnover within the FHS, training and educational background, and the motivation of oral health professionals.

## Data Availability

The datasets generated during and/or analyzed during the current study are available from the corresponding author on reasonable request.
